# Acute Myocardial Infarction and Diffuse Coronary Artery Disease in a Patient with Multiple Sclerosis: A Case Report and Literature Review

**DOI:** 10.3390/jcm14124304

**Published:** 2025-06-17

**Authors:** Eugen Nicolae Țieranu, Silvana Isabella Cureraru, Georgică Costinel Târtea, Viorel-Cristian Vladuțu, Petre Alexandru Cojocaru, Mina Teodora Luminița Piorescu, Loredana Maria Țieranu

**Affiliations:** 1 Department of Cardiology, Emergency County Hospital, 200642 Craiova, Romania; eugen.tieranu@umfcv.ro (E.N.Ț.); isabellacureraru@yahoo.ro (S.I.C.); george.tartea@gmail.com (G.C.T.); vladutu.cristian@yahoo.com (V.-C.V.); 2 Department of Cardiology, University of Medicine and Pharmacy of Craiova, 200349 Craiova, Romania; 3 Department of Physiology, University of Medicine and Pharmacy of Craiova, 200349 Craiova, Romania; 4 Department of Dermatology, Emergency County Hospital, 200642 Craiova, Romania; 5 Department of Obstetrics and Gynaecology, Emergency County Hospital, 200642 Craiova, Romania; loredanapospai@yahoo.com

**Keywords:** multiple sclerosis, acute coronary syndrome, ST-elevation myocardial infarction, percutaneous coronary intervention, cardiovascular risk

## Abstract

Multiple sclerosis (MS) is a chronic progressive neurodegenerative disease that leads to disabilities such as difficulty moving and slowed cognitive processing. It is the leading non-traumatic cause of disability worldwide. MS also has a high potential to become a model for neurodegenerative diseases with a progression like Alzheimer’s or Parkinson’s. Cardiovascular diseases (CVDs) remain the leading cause of global deaths and have a considerable economic impact. The higher incidence of cardiovascular comorbidities in patients with MS compared to healthy individuals of the same age worsens the prognosis of neurological pathology, leading to a higher level of disability, poorer physical outcomes, higher depression scores, cognitive aging, and diminished quality of life. Classical observational studies often have questionable elements that can represent a source of error, making it difficult to establish a causal relationship between MS and CVD. Genetic studies, including genome-wide evaluation, may resolve this issue and may represent a topic for future research. We report the case of a 31-year-old male patient with a history of multiple sclerosis (MS) diagnosed seven years prior, who presented with acute chest pain upon returning from vacation. Despite the previous recommendation for disease-modifying therapy, the patient had discontinued treatment by personal choice. Electrocardiography (ECG) revealed ST-segment elevation in inferior leads, and emergent coronary angiography identified severe multi-vessel coronary artery disease (CAD), requiring immediate revascularization. This case highlights the potential cardiovascular risks in young patients with MS and the importance of continuous medical supervision.

## 1. Introduction

Ischemic heart disease remains a public health problem [1], as it affects a very large number of people and the costs are very high both in terms of acute-phase treatment and long-term treatment. Although the elderly are most frequently affected, the incidence of coronary heart disease in recent years has been continuously increasing in younger age groups [2,3]. Although conventional risk factors are present in young patients with coronary artery disease, such as lifestyle, obesity, dyslipidemia and smoking [4], there is a fundamental difference that underlies our research, namely, the significantly shorter exposure time. This led us to believe that there must be other risk factors that have not been sufficiently investigated in young people. There are recent studies that have highlighted the presence of some risk factors considered new, which are involved in the occurrence of acute myocardial infarction with ST segment elevation in young people [5], but whose mechanism needs to be further investigated. Among the factors considered non-traditional in young patients with acute myocardial infarction, we can note drug use, chronic inflammation [5], autoimmune diseases and hypercoagulability [6,7].

There is a wealth of literature comparing conventional risk factors in young versus older adults. The most common conventional risk factor identified in young adults was smoking [8]. There are also numerous studies that have shown that in young patients with angiographically assessed coronary artery disease, the prevalence of dyslipidemia dislipidemiei [9], obesity [10] and hypertension [11] was significantly lower than in the older groups, this observation being very important. On the other hand, diabetes mellitus was not considered an important risk factor for myocardial infarction in young people under 40 years of age [12].

In this research we focused our attention on identifying and understanding some less studied mechanisms, such as the autoimmune mechanism involved in the pathophysiology of multiple sclerosis [13], and on the role they have in determining myocardial infarction, especially in young people without conventional risk factors. Starting from these considerations, we tried to identify in the specialized literature a relationship between the presence of multiple sclerosis and the development of an acute coronary syndrome.

Worldwide, over two million people suffer from MS [14]. These patients are being treated with glucocorticoids, usually administered in high doses, and this may be a factor that causes an accelerated atherosclerotic process [15]. In addition to the direct effect of glucocorticoids, they are predisposed to elevated glycemic values, with a substantial risk of developing iatrogenic diabetes mellitus, and also to a high incidence of obesity [16]. Another suggestive element that may underlie the accelerated atherosclerotic process and CAD in patients with MS is the higher incidence of tobacco consumption compared to the general population [16,17].

### 1.1. The Role of Inflammation

Atherosclerosis is a sustained and evolving process in which the immune system plays a central role. In an effort to better understand the link between inflammation and cardiovascular disease, we examined the existing body of literature.

The hematogenous marrow plays an essential role in this process, being responsible for the synthesis of monocytes, which subsequently migrate to various tissues and differentiate into macrophages [18]. Depending on local and environmental stimuli, these macrophages can acquire a pro-inflammatory (M1) or anti-inflammatory (M2) phenotype [19]. In addition to factors such as lipopolysaccharides, interferon-gamma and pro-inflammatory cytokines—produced mainly by T lymphocytes and natural killer cells—the activation of the M1 phenotype is strongly influenced by changes in cellular metabolism, especially by increased anaerobic glycolysis [20]. It has been demonstrated that macrophages activated by LPS have an increased expression of SLC2A1/GLUT-1, associated with increased glucose uptake and, implicitly, the glycolysis process [21]. The glycolysis process in M1 is mediated mainly by the increased expression of PKM and PKM2, which are glycolytic enzymes [22]. Also, glucocorticoids, widely used in the treatment of various conditions, significantly contribute to the activation of M1 macrophages [23]. In recent years, a distinct type of macrophage, called MOX, has been described, which has been identified in mice in atheromatous plaques in the aorta [19]. MOX was subsequently shown to represent a more pathogenic M1 phenotype associated with accelerated atherosclerosis, which appears to be induced by elevated levels of oxLDL [24]. In addition to LDL, which stimulates the hematogenous marrow in the production of monocytes, cytokines such as CCL2/MCP-1, which binds the CCR2 receptor and facilitates the migration of monocytes to the lesion, also play an important role [25]. Macrophage activity has also been studied in young MS patients. Immunohistochemical studies have shown increased uptake of the MRP14 marker in acute inflammatory phases, while the 27E10 marker has been identified in chronic inflammatory phases in MS patients [26,27]. Moreover, it has been demonstrated that the MOX phenotype predominantly binds the MRP14 marker [28].

In addition to monocytes, there are other elements that play a role in the pathogenesis of atherosclerosis. Tumor necrosis factor-α, interleukin 1β (IL-1β) and IL-6 play an important role in the pathophysiology of atherosclerosis, from the onset of endothelial dysfunction to plaque rupture. Also, activation of the nucleotide-binding oligomerization domain-like receptor family [29] and the pyrin domain-containing protein 3 inflammasome appear to be associated with the development and progression of atherosclerotic cardiovascular disease [30]. In addition to inflammation, the oxidative process causes that play a role in the complex process of atherosclerosis, including lipoproteins, secretory phospholipase A2, mitogen-activated p38 kinase, and P-selectin [31].

The participation of immune cells—T cells, B cells, but also microglia resident in the CNS—have a defining role in the pathogenesis of MS [32]. Inflammatory processes encountered in MS patients are identified in both the peripheral and central compartments. Inflammation caused by the activation of these cells is responsible for the alteration of the blood—brain barrier (BBB) and the transendothelial migration of activated leukocytes, leading to the loss of oligodendrocytes and neuro-axonal damage with the final effect of CNS damage. Macroscopically, the process is focal, occurring throughout the CNS (brain, optic nerve and spinal cord), involving both white matter and gray matter [33]. This chronic proinflammatory state found in MS patients can accelerate the process of atherosclerosis [34].

On the other hand, multiple sclerosis and myocarditis share similar molecular and immune mechanisms, being inflammatory diseases affecting the central nervous system and the heart. Both are focal diseases with characteristic lesions and benefit from a certain immunological privilege. In MS, the initial phases are marked by demyelinating plaques with T and B cell infiltrates, microglial activation, and oligodendrocyte loss [35]. Chronic lesions occur in patients with progressive forms. In myocarditis, the initial inflammation involves T cells (CD3+) and myocyte necrosis, which may progress to fibrosis, dilated cardiomyopathy, or may resolve completely [36]. Both diseases involve Th1, Th17 and Th2 cells, and dysregulation of the Treg/Th17 balance is a common feature, suggesting possible therapeutic targets. B cells are involved in both pathologies, but specific antibodies are more clearly associated with myocarditis than with MS [37].

At the same time, the role of infectious factors should not be minimized. The coronavirus pandemic highlighted that cardiovascular diseases were present in up to 16.4% of patients with COVID-19, and the presence of these comorbidities significantly increased the risk of coronary heart disease and death [38]. A plausible explanation for these consequences is the inflammatory process, showing that SARS-CoV-2 is involved in the occurrence of acute coronary syndromes through systemic inflammatory activation and cytokine release [38]. This identifies a critical need for long-term study of patients with COVID-19 to understand the cardiovascular sequelae of this disease [38]. SARS-CoV-2 is also linked to cases of myocarditis, and some infections or COVID-19 vaccines may trigger the onset of MS or exacerbate existing symptoms [39,40]. Not only SARS-CoV is associated with MS and myocarditis, but also Epstein–Barr virus (EBV) is one of the most intensively studied pathogens in the context of T-cell responses in multiple sclerosis (MS). EBV viral antigens can induce the development of interleukin-10 (IL-10)-producing CD4+ regulatory T cells (Tregs), capable of suppressing T-cell responses upon re-exposure to the antigen [41]. For example, in MS patients, Treg populations are often enriched but exhibit increased levels of interferon-gamma (IFN-γ), reduced expression of the transcription factor FOXP3 and impaired suppressive capacity in vitro [42]. FOXP3 correlates with both IL-2 receptor (IL-2R) and IL-7 receptor (IL-7R) expression in Treg cells [43]. Therefore, it is hypothesized that genetic variants in the IL2RA and IL7RA genes [44,45], as well as BACH2 [46], may influence Treg development and function in MS. This dysfunction may also affect CD8+ T cells expressing FOXP3 and IL-2R, which are known to suppress proinflammatory CD4+ helper T cells [45] and are significantly reduced in the circulation during MS relapses [47]. In myocarditis, viruses can cause persistent inflammation, including EBV, which has been identified in cases of acute and chronic myocarditis [48]. Although not frequently reported together, MS and myocarditis have common mechanisms and may be influenced by the same viral and immunological factors, especially in individuals with genetic susceptibility.

There is currently sufficient evidence to show that postulated immune system dysfunctions may represent risk factors for the future development of cardiovascular disease through fibrosis or accelerated atherosclerosis. A significant factor in the alteration of immune mechanisms is insulin resistance, and an increased HOMA-IR index above the normal limit can also be caused by hepatitis C virus infection [49], as observed in our patient. Dysfunction of the immune system and alteration of inflammatory pathways may represent a very important risk factor for coronary heart disease, especially in young patients, which should be considered when diagnosed, especially if the patients are known to have systemic and long-term inflammatory pathologies.

In our study, we focused on multiple sclerosis and on the possible associations with coronary heart disease. The most important risk factors implicated in the onset of multiple sclerosis indicate that low serum vitamin D levels, smoking, childhood obesity and Epstein-Barr virus infection are likely to play a role in the development of the disease [50]. Multiple sclerosis (MS) begins well before the appearance of clinical signs—even 10–20 years earlier—although asymptomatic MS can be detected by performing an occasional MRI for nonspecific symptoms such as headaches, head trauma, or screening in the airline industry [51].

### 1.2. Multiple Sclerosis and MI—Two Similar Pathologies

Multiple sclerosis is a chronic neurological condition of the central nervous system, characterized by walking difficulties and slowing of cognitive processing, and is the main cause of non-traumatic disability, with a significant impact on the quality of life of affected people [52,53]. Autonomic nervous system (ANS) dysfunction is common in multiple sclerosis (MS) and affects various functions—cardiovascular, urinary, sexual, bowel and sweat. Up to two-thirds of MS patients have cardiovascular autonomic dysfunction, associated with brain damage or spinal atrophy. This dysfunction can contribute to heart rhythm disturbances and an increased risk of cardiovascular disease [54].

In recent years, an increasingly close relationship between MS and coronary heart disease has been intensively studied through epidemiological research. Thus, there is evidence that patients with multiple sclerosis have an increased risk of acute coronary syndrome [54]. These patients not only have a higher cardiovascular risk but also a higher incidence of cerebrovascular disease, ischemic stroke being frequently encountered [55]. On the other hand, MS patients from the Nordic population are associated with a low risk of atrial fibrillation [56].

Recently, in 2022, genetic evaluations covering 48% of the heritability of MS were performed, including 47,429 MS patients and 68,374 controls, and identified 233 single nucleotide polymorphisms (SNPs), of which 68 were associated with MS. The results of the mendelian randomization (MR) analysis based on the inverse variance-weighted (IVW) method showed that MS may be involved in the occurrence of myocardial infarction (MI) (OR = 1.044, 95% CI: 1.015–1.074, *p* = 0.002) and atrial fibrillation (AF) (OR = 1.034, 95% CI: 1.007–1.061, *p* = 0.012). However, after Bonferroni correction (*p* < 0.006), only the association with MI appears to have statistical significance, without MI being involved in the occurrence of MS [57].

The identification of genetic factors allowed the discovery of the MHC2TA gene, which was associated with both AMI and MS. Although a polymorphism of MHC2TA, 168G/A is certainly implicated in the occurrence of SM and rheumatoid arthritis. A detailed study of this gene identified a correlation between a mutation in this gene and a higher frequency of both AMI and MS; it is difficult to understand at this time its role in the occurrence of AMI and whether the association is due more to the inflammatory process [58]. The development of molecular genetics in recent years has allowed identifying a very large number of genes involved in the pathogenesis of the two pathologies; more precisely, 2377 genes met the criteria for upregulation of AMI, and 180 genes met the criteria for upregulation of MS. Through the analysis of overlaps, a gene involved in the IL-17 signaling pathway was identified, with a high probability of being part of a large mechanism behind the relationship between MS and AMI, which gives credibility to the genetic theory [59].

The processes by which cardiovascular risk factors are involved in the development of MS remain elusive. It has been observed that even in the absence of cerebrovascular disease, diabetes, dyslipidemia and hypertension are associated with brain atrophy [60], and peripheral inflammation seems to play a dominant role [61]. In another vein, abnormalities of cerebral endothelial cells are reported in MS and may be amplified by cardiovascular risk factors [62]. Thus, it is possible that multiple mechanisms contribute to explaining the interactions between multiple cardiovascular comorbidities and MS.

Although there is data that seems to outline pathophysiological links between the two pathologies, there are also many things that are not fully elucidated, both from genetic factors, classic and new risk factors, and the mechanisms by which environmental and infectious factors can influence the immune system.

### 1.3. MS Treatment and Cardiovascular Side Effects

Although MS is a well-studied disease and a health problem of interest, there is still no prophylactic therapy or curative treatment for it. Patients with MS suffer from a series of disabilities that make their lives difficult, so treatment remains aimed at preventing disabilities and slowing the progression of the disease [63]. Disease-modifying therapies (DMTs) can influence the course of MS by regulating the immune response and reducing inflammation, especially during relapses. However, MS and some associated treatments can affect the cardiovascular system, increasing the risk of severe complications [64].

A variety of molecules that prove to be useful in the treatment of multiple sclerosis have been studied over time. One of these is fingolimod, whose mechanism of action is by blocking sphingosine-1-phosphate receptors. A notable adverse effect is bradycardia and the risk of AV block, which requires an ECG and blood pressure measurement [63,64,65]. Another treatment that has proven useful in multiple sclerosis, but not without cardiotoxic effects, is mitoxantrone, an immunomodulatory agent, which, according to Kaplan et al., has an increased incidence of heart failure in patients treated with high cumulative doses [64].

Drugs such as rituximab, which, through their mechanism, have a favorable effect on cardiac function in terms of improving ejection fraction, nevertheless present cardiotoxic potential according to newer data, with effects such as arrhythmias, cardiogenic shock, hypotension and even myocardial infarction being cited during the infusion process [66].

On the other hand, there are drugs, such as dimethyl fumarate, with protective effects on the heart, especially by reducing the risk of myocardial injury [67].

In MS patients, there is an increased risk of bleeding due to immunosuppressive treatments that may affect platelet function and hemostasis. Triple anticoagulant therapy, as used in this case (Aspirin, Plavix, and Rivaroxaban) [68], may increase the risk of major bleeding, especially in the context of liver dysfunction associated with HCV infection. Additionally, MS patients may have an increased risk of intracerebral bleeding, and anticoagulants can complicate these events, given the associated cerebrovascular conditions.

## 2. Case Report

**Figure 1:**
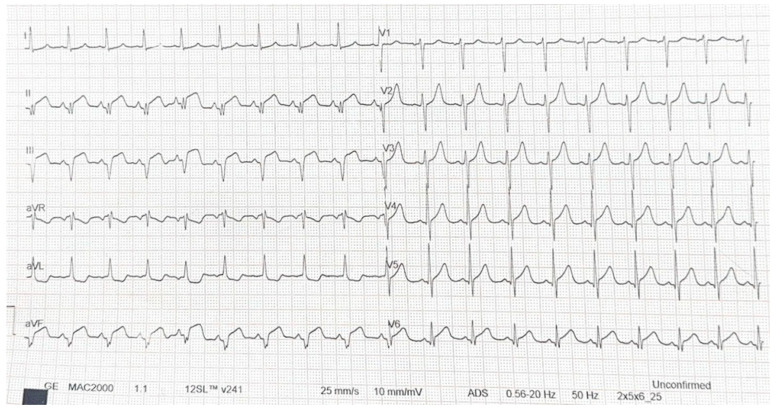
Emergency ECG performed.

A 31-year-old male, diagnosed with MS seven years earlier, presented with prolonged anterior chest pain radiating to the neck while at the airport. The pain did not subside with rest. Approximately two hours after symptom onset, the patient arrived at the emergency department, where ECG showed ST-segment elevation in leads DII, DIII, AVF, V5, V6 (Figure 1).

Laboratory findings included the following: Elevated myocardial necrosis markers: CK 553U/L, CK-MB 72U/L, TNT-HSST 1047 pg/mL; Erythrocyte sedimentation rate: 64 mm/h; C-reactive protein: 43.54 mg/L; HOMA-IR: 2.5; Hepatitis C virus positive; LDL cholesterol: 190 mg/dL; Thrombophilic protein C: 162%

Echocardiographic findings: The left ventricle is not dilated; left ventricular systolic function is preserved; however, there is hypokinesia in the basal two-thirds of the inferior wall, the apical one-third of the anterior wall, the basal two-thirds of the anterior septum, and the basal one-third of the posterior wall; mild mitral and tricuspid regurgitation; spontaneous contrast observed in the left ventricle.

**Figure 2:**
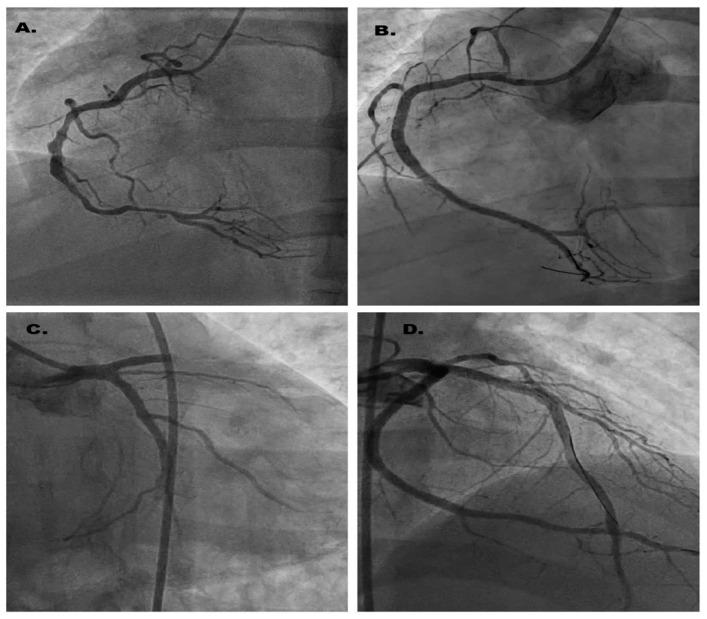
Illustration of coronary images captured during the two revascularization procedures. (**A**) Ulcerated 90% plaque in segment III of the RCA. (**B**) Right coronary artery after revascularization. (**C**) TIMI 0 distal flow in LAD and LCx. (**D**) LCx after revascularization and TIMI 3 distal flow.

**Figure 3:**
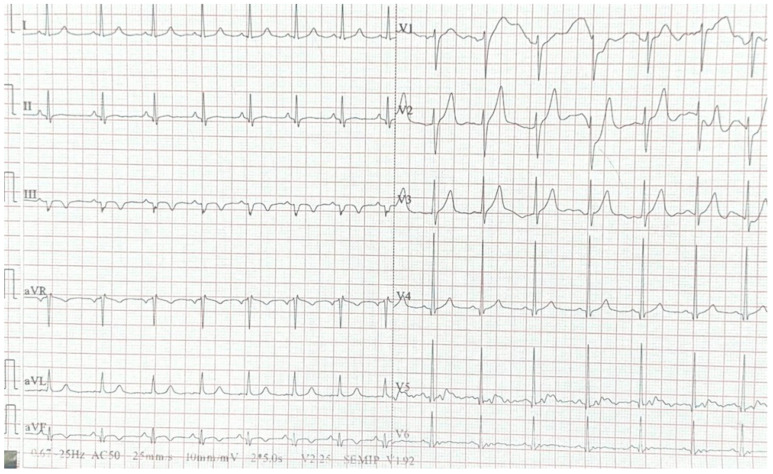
ECG performed after RCA revascularization.

Emergency coronary angiography identified: Two serial 80% lesions in segments I-II of the right coronary artery (RCA) and an ulcerated 90% plaque in segment III of the RCA (Figure 2A). A subocclusive stenosis at the apex of the left anterior descending artery (LAD). A 70% stenosis in the mid-to-distal segment of the left circumflex artery (LCx). In conclusion, the patient presents with three-vessel coronary artery disease. Considering ECG changes and the presence of coronary lesions, we decided to perform angioplasty on the right coronary artery (RCA) with the implantation of three drug-eluting stents: 2.5 × 18 mm, 2.75 × 38 mm, and 3.0 × 28 mm (Figure 2B), from the distal to the proximal segment. After RCA revascularization, the resolution of ST-segment elevation is observed on the ECG (Figure 3).

**Figure 4:**
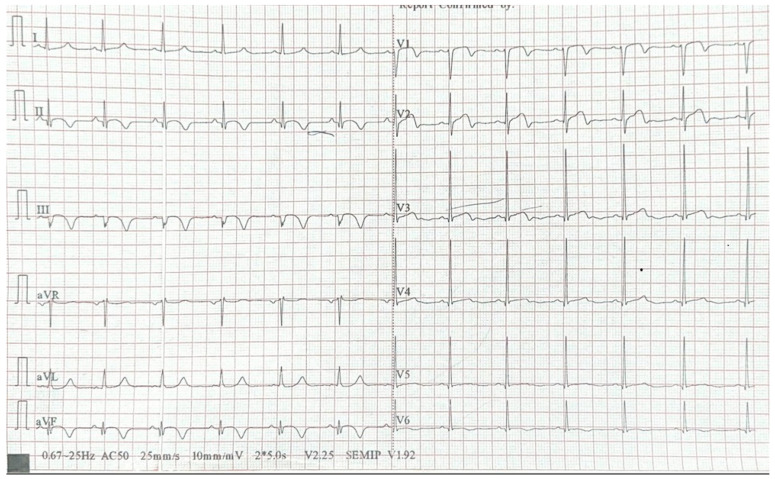
ECG after the second procedure.

During the second revascularization procedure of the left circumflex artery (LCx), performed under unfractionated heparin treatment (100 IU/kg), an acute, possibly thrombotic occlusion was observed after the first injection, affecting both the left anterior descending artery (LAD) and the LCx, with TIMI 0 distal flow (Figure 2C), followed by hemodynamic deterioration. Immediately, two PCI guidewires were placed in the distal segments of the LAD and LCx, restoring flow, after which a 3.0 × 48 mm drug-eluting stent was implanted and expanded at 14 atm (Figure 2D). After the second revascularization, a Q wave and negative T waves are observed in the inferior territory, along with minimal ST-segment elevation and negative T waves in the anterior territory (Figure 4).

Considering the aforementioned factors and the association between acute coronary syndrome and thrombophilic pathology, it was decided to administer triple antithrombotic therapy for 30 days with Aspirin 75 mg, Plavix 75 mg and Rivaroxaban 20 mg. After this period, the patient was prescribed Clopidogrel 75 mg and Rivaroxaban 20 mg for up to one year. Additionally, he received Rosuvastatin/Ezetimibe 40/10 mg, Perindopril 5 mg, Spironolactone 50 mg, Metoprolol succinate 50 mg, and Pantoprazole 20 mg.

The patient was also advised to undergo a neurological evaluation for the initiation of specific treatment for multiple sclerosis, as well as a gastroenterological evaluation.

## 3. Discussion

In this article, we have attempted to trace the complex relationship between multiple sclerosis and ischemic heart disease, especially myocardial infarction, with an emphasis on inflammatory mechanisms and immune dysfunction. Although traditional cardiovascular risk factors, such as smoking, dyslipidemia, or hypertension [69], are frequently encountered among young people with IHD, there is a category of patients in whom these factors are absent [70]. This observation has generated the hypothesis of the existence of alternative or complementary mechanisms, insufficiently explored to date.

The pathophysiology of MS is largely a chronic inflammatory process in which immune cells (T, B, microglia) [71], monocytes and the secretion of proinflammatory cytokines, such as IL-6 or TNF-α, play a very important role [72]. This exacerbated immune activity can cause an accelerated atherosclerotic process, thus defining the links between the two entities. In addition to the atherosclerotic process, MS, which is characterized by characteristic autonomic dysfunctions, can cause arrhythmias and cardiovascular dysfunction, thus increasing the risk of death in patients with MI [73]. However, current data remain limited, with the relationship between the two pathologies being more of a research topic than a certainty but with a valid basis.

On the other hand, MS treatment is associated with an increased risk of cardiotoxicity, such as glucocorticoids or immunomodulatory therapies. Modern therapies tend to reduce cardiovascular risk and even have a protective effect on the heart, such as dimethyl fumarate [74]. However, it is very important to monitor cardiac function in patients treated for MS.

Genetic studies have identified similarities between MS and MI involving common inflammatory pathways such as IL-17 [75], and the identification of the MHC2TA gene as a common point between the two pathologies strengthens the hypothesis of a predisposing genetic substrate [58], not yet fully elucidated.

From another perspective, we cannot deny the obvious: the case presented emphasizes a significant association between multiple sclerosis (MS) and cardiovascular risk, demonstrating that patients with neuroinflammatory diseases may develop major cardiovascular events at a young age. The 31-year-old patient presented in this report suffered an acute myocardial infarction with ST-segment elevation (STEMI), having multiple significant coronary lesions, although he did not present major conventional cardiovascular risk factors such as hypertension or diabetes.

Chronic inflammation and immune dysfunction—Systemic inflammation plays a key role in atherosclerosis, and the presence of MS may exacerbate this process through inflammatory and autoimmune mechanisms. Elevated CRP and ESR levels observed in this patient suggest an active inflammatory process [76].

Comorbidities—Hepatitis C virus (HCV) infection may contribute to vascular damage through pro-inflammatory and pro-thrombotic mechanisms. Additionally, high LDL cholesterol levels indicate increased metabolic risk [77].

Acute thrombotic event—The intra-procedural evolution, with acute possibly thrombotic occlusion of the coronary arteries, highlights an increased risk of thrombosis in these patients. The choice of triple antithrombotic therapy reflects this susceptibility [78].

Discontinuation of MS treatment—The lack of disease-modifying therapy for MS may be an aggravating factor in the systemic inflammatory process and could indirectly contribute to endothelial dysfunction and increased cardiovascular risk [79].

Although the vast majority of what we know about acute myocardial infarction does not apply to our patient, there are certainly other elements that are perhaps less known or researched that must be addressed in young patients and those with associated inflammatory or autoimmune pathologies in order to prevent certain complications.

## 4. Conclusions

The relationship between MS and BCI, especially MI, is a bidirectional one and is influenced by immunological, genetic, infectious and therapeutic factors, even though the interaction of these factors is still unclear. Our study contributes to a better understanding of cardiovascular risk among young patients with MS and highlights the need for a multidisciplinary and personalized approach to prevent cardiovascular complications in this vulnerable group.

Patients with multiple sclerosis face an elevated cardiovascular risk, even in the absence of traditional risk factors, highlighting the importance of thorough cardiovascular monitoring. The chronic systemic inflammation and immune dysregulation associated with MS may contribute to the acceleration of atherosclerosis and increase the likelihood of thrombotic events. To ensure optimal treatment and effective secondary prevention, a multidisciplinary approach is essential, bringing together neurologists, cardiologists and infectious disease specialists to address the complex needs of these patients.

Although we cannot draw a direct link between AMI and MS, there are certainly intricate mechanisms that can facilitate the occurrence of the two pathologies, but these mechanisms are very complex and poorly studied.

## 5. Future Directions

Globally, there is a relatively small group of young patients with multiple sclerosis who develop acute cardiovascular events and do not have other associated risk factors, which may represent a limitation of this research.

There are numerous genetic studies in the research that aim to reduce cardiovascular risk by identifying and treating cardiovascular risk factors early, such as hypercholesterolemia, hypertension, hypercoagulability, predisposition to obesity and diabetes, thus enabling early interventions from a young age for prompt correction and avoidance of acute events.

Also, modern approaches in treating inflammatory diseases through biological therapies have demonstrated significant improvement over conventional glucocorticoid treatments, with side effects that appear acceptable.

## Data Availability

All data is available upon request.

## Abbreviations

The following abbreviations are used in this manuscript:

ECG	Electrocardiogram
LAD	Anterior descending artery
LCX	Left circumflex artery
RCD	Right coronary artery
MS	Multiple sclerosis
CVD	Cardiovascular disease
CAD	Coronary artery disease
MI	Myocardial infarction
